# Chimpanzee Malaria Parasites Related to *Plasmodium ovale* in Africa

**DOI:** 10.1371/journal.pone.0005520

**Published:** 2009-05-13

**Authors:** Linda Duval, Eric Nerrienet, Dominique Rousset, Serge Alain Sadeuh Mba, Sandrine Houze, Mathieu Fourment, Jacques Le Bras, Vincent Robert, Frederic Ariey

**Affiliations:** 1 Laboratoire de Biologie fonctionnelle des protozoaires, USM 504, Muséum National d'Histoire Naturelle, Paris, France; 2 Laboratoire de Pathogénie virale, Institut Pasteur, Paris, France; 3 Laboratoire HIV et Hepatites, Institut Pasteur du Cambodge, Phnom Penh, Cambodia; 4 Unité de virologie, Centre Pasteur du Cameroun, Yaoundé, Cameroun; 5 Centre National de Référence du Paludisme, AP-HP, Hôpital Bichat-Claude Bernard, Paris, France; 6 Unité de Virologie, Institut Pasteur du Cambodge, Phnom Penh, Cambodia; 7 Department of Biological Sciences, Macquarie University, Sydney, Australia; 8 Unité de Recherche Caractérisation et contrôle des populations de vecteurs, UR 16, Institut de Recherche pour le Développement, Montpellier, France; 9 Unité d'Epidémiologie Moleculaire, Institut Pasteur du Cambodge, Phnom Penh, Cambodia; Université Pierre et Marie Curie, France

## Abstract

Since the 1970's, the diversity of *Plasmodium* parasites in African great apes has been neglected. Surprisingly, *P. reichenowi*, a chimpanzee parasite, is the only such parasite to have been molecularly characterized. This parasite is closely phylogenetically related to *P. falciparum*, the principal cause of the greatest malaria burden in humans. Studies of malaria parasites from anthropoid primates may provide relevant phylogenetic information, improving our understanding of the origin and evolutionary history of human malaria species. In this study, we screened 130 DNA samples from chimpanzees (*Pan troglodytes*) and gorillas (*Gorilla gorilla*) from Cameroon for *Plasmodium* infection, using *cytochrome b* molecular tools. Two chimpanzees from the subspecies *Pan t*. *troglodytes* presented single infections with *Plasmodium* strains molecularly related to the human malaria parasite *P. ovale*. These chimpanzee parasites and 13 human strains of *P. ovale* originated from a various sites in Africa and Asia were characterized using *cytochrome b* and *cytochrome c oxidase 1* mitochondrial partial genes and nuclear *ldh* partial gene. Consistent with previous findings, two genetically distinct types of *P. ovale*, classical and variant, were observed in the human population from a variety of geographical locations. One chimpanzee *Plasmodium* strain was genetically identical, on all three markers tested, to variant *P. ovale* type. The other chimpanzee *Plasmodium* strain was different from *P. ovale* strains isolated from humans. This study provides the first evidence of possibility of natural cross-species exchange of *P. ovale* between humans and chimpanzees of the subspecies *Pan t. troglodytes*.

## Introduction

*Plasmodium ovale*, *P. falciparum*, *P. vivax* and *P. malariae* belong to phylum Apicomplexa, order Haemosporidia and family Plasmodiidae. Haemosporidia are intracellular parasites transmitted by haematophagous dipterans. They infect a large variety of vertebrate amniotes, such as mammals (including humans), birds, chelonians, squamates, and crocodilians, [1]. Some are highly pathogenic and may have important implications for human public health, domestic animal health and wildlife biodiversity conservation [2], [3].

*P. ovale*, the last of the human malaria parasites to be identified, was described in the blood of an East African patient, by Stephens in 1922. It is a relapse parasite, generating secondary infections that are usually asymptomatic [4]. However, *P. ovale* may interact with other species of *Plasmodium* infecting humans, such as *P. falciparum* and *P. vivax*, and may have a major influence on the epidemiological features of malaria [5].

Few epidemiological data are available for *P. ovale*. Its reported prevalence is generally low (<5%), except in West Africa, where prevalences above 10% have been observed in humans [6], [7]. *P. ovale* is often present in mixed infections and parasitaemia is usually low.

*P. ovale* was previously thought to be present only in sub-Saharan Africa, Papua New Guinea, Irian Jaya in Indonesia and the Philippines [4]. However, it appears to be more widely distributed, having been reported in the Middle East, the Indian Subcontinent and various parts of Southeast Asia [8]–[11]. *P. ovale* has not been yet reported in South America. However, no global map of the geographical distribution of *P. ovale* has been produced since that of Lysenko and Beljaev in 1969 [12].

Few studies document the molecular diversity, geographical origin, evolutionary history and age of *P. ovale* populations. Based on complete DNA sequences of the small subunit ribosomal RNA (*SSUrRNA*) gene, partial sequences of cysteine protease, ookinete surface protein and *cytochrome b* genes, Win et al. (2004) compared *P. ovale* isolates from Myanmar, Indonesia and sequences available from GenBank. The result obtained supported the division of *P. ovale* into at least two types, but the classical and variant types identified did not differ morphologically and occurred in sympatry [13], [14].

Phylogenetically, *P. ovale* clusters with *Plasmodium* species affecting simian primates (as do *P. malariae* and *P. vivax*, but not *P. falciparum*), but its phylogenetic relationships to other *Plasmodium* species or haemosporidian parasite genera remain unclear [4].

Three *Plasmodium* species, *P. reichenowi*, *P. schwetzi* and *P. rodhaini*, have already been reported in African great apes (chimpanzees and gorillas) and have been described as morphologically similar to *P. falciparum*, *P. ovale* or *P. vivax* (there are differing opinions) and *P. malariae*, respectively [15]. Like humans, the African great apes belong to the Hominidae family. Despite the close phylogenetic relationships between these non human primates and human hosts, the diversity of *Plasmodium* parasites in African great apes has been little studied and few molecular data for these parasites are available. Indeed, only one strain of *P. reichenowi*, originally isolated from a naturally infected chimpanzee (*Pan troglodytes*) in Central Africa (East of the Democratic Republic of the Congo) and adapted to a laboratory splenectomized chimpanzee, has been molecularly characterized [15]. This parasite is closely phylogenetically related to *P. falciparum*, the principal cause of human malaria. Data for other taxa, including genetically characterized non human primate malaria parasites, are required to provide insight into the evolutionary history of *P. ovale* [16].

In order to investigate the diversity of *Plasmodium* parasites in African great apes, we screened 130 DNA samples from chimpanzees and gorillas in Cameroon. We found three chimpanzees infected by *Plasmodium* related to the human *P. ovale*. We present here the diversity of these chimpanzee parasites using two mitochondrial and one nuclear partial gene sequences and compared them to human *P. ovale* strains.

## Results

DNA samples from 130 chimpanzees and gorillas were tested for *Plasmodium* infection, using *cytochrome b* molecular tools. Two chimpanzees, CPZcam89 (225) and CPZcam91 (451), both belonging to subspecies *Pan t*. *troglodytes*, presented a single infection with *Plasmodium* parasites phylogenetically related to *P. ovale*. Both *Plasmodium* isolates were characterized by a unique DNA sequence for each of the *cox1*, *cyt b* and *ldh* markers, differing between the two isolates. A third chimpanzee (CPZcam63 (2360)), belonging to subspecies, *Pan t*. *vellerosus*, had a mixed infection composed of *P. reichenowi* and *P. ovale* related parasites. The latter has an identical *cyt b* sequence to *Plasmodium* found in CPZcam89 (451) chimpanzee; this isolate was discarded from the phylogenetic construction. The prevalence of *P. ovale* related *Plasmodium* species was found to be 2.3% (3/130) in the Cameroonian great apes tested. This prevalence is comparable to the prevalence of *P. ovale* in human populations from most endemic areas (<5%).

The 708 bp *cyt b* and the 964 bp *cox1* sequences as well as the 350 bp *ldh* sequence of the CPZcam89 (225) chimpanzee parasite strain are all identical to the human *P. ovale* variant type sequences (Tables 1, 2 and 3). Based on this genetic homology, this chimpanzee parasite strain was identified as being of the *P. ovale* variant type. The *cyt b*, *cox1* and *ldh* nucleotide sequences of the CPZcam91 (451) chimpanzee parasite diverged from the reported classical and variant *P. ovale* type nucleotide sequences (Tables 1, 2 and 3). For the *cyt b* marker, this chimpanzee *Plasmodium* sequence presented four synonymous mutations with respect to the classical *P. ovale* type sequence and one non synonymous mutation, M248I, with respect to the variant *P. ovale* type sequence (Table 1). The *cox1* marker displayed two non synonymous mutations with respect to the classical *P. ovale* type and three with respect to the variant *P. ovale* type (Table 2). The nuclear *ldh* sequence shows two non synonymous mutations compared to the classical *P. ovale* and four non synonymous mutations compared to the variant *P. ovale* (Table 3).

**Table 1:** Substitutions and their positions in *cyt b* nucleotide sequences (numbers correspond to base pair positions and were defined according to the complete *P. falciparum cyt b* gene sequence M76611).

Sequences	315	375	402	450	492	510	514	534	744	756	774	885	903	948
*P. ovale* classical type	-	-	-	-	-	-	-	-	-	-	-	-	-	-
*P. ovale* variant type	-	A	T	-	A	-	-	T	T	T	A	T	T	A
									(M248I)					
CPZcam89 (225)	-	A	T	-	A	-	-	T	T	T	A	T	T	A
									(M248I)					
CPZcam91 (451)	A	-	-	A	-	T	T	-	-	-	-	-	-	-

Non synonymous mutation is shown in brackets.

**Table 2:** Substitutions and their positions in *cox1* nucleotide sequences (numbers correspond to base pair positions and were defined according to the complete sequence of the *P. falciparum cox1* gene M76611).

Sequences	449	458	462	473	575	632	650	657	761	764	765	766	830	966	1016	1022	1082
*P. ovale* classical type	-	-	-	-	-	-	-	-	-	-	-	-	-	-	-	-	-
*P. ovale* variant type	C	C	T	T	G	A	-	T	-	-	-	-	T	G	-	-	A
						(M211I)											
CPZcam89 (225)	C	C	T	T	G	A	-	T	-	-	-	-	T	G	-	-	A
						(M211I)											
CPZcam91 (451)	-	-	T	-	-	-	G	-	A	T	A	C	-	-	A	T	A
										(L255F)	(H256T)						

Non synonymous mutations are shown in brackets.

**Table 3:** Substitutions and their positions in *ldh* nucleotide sequences (numbers correspond to base pair positions and were defined according to the complete sequence of the *P. falciparum ldh* gene PF13_0141).

Sequences	195	237	243	258	291	301	321	333	337	339
*P. ovale* classical type	-	-	-	-	-	-	-	-	-	-
*P. ovale* variant type	C	A	C	A	-	C	C	-	-	T
										
CPZcam89 (225)	C	A	C	A	-	C	C	-	-	T
										
CPZcam91 (451)	-	-	-	-	T	-	-	T	G	-
									(I113V)	

Sequences	351	361	387	406	427	475	504	507	510
*P. ovale* classical type	-	-	-	-	-	-	-	-	-
*P. ovale* variant type	-	-	-	C	C	T	G	A	C
					(S143P)		(K168N)		
CPZcam89 (225)	-	-	-	C	C	T	G	A	C
					(S143P)		(K168N)		
CPZcam91 (451)	T	C	T	-	-	-	-	G	C
		(F121L)							

Non synonymous mutations are shown in brackets.

Investigation of the mitochondrial *cyt b*, *cox1* and nuclear *ldh* partial gene sequences in 13 *P. ovale* strains from humans from 12 different sites showed that *P. ovale* species could be divided into two distinct groups. Both classical and variant *P. ovale* (Table 4) were associated with a unique sequence for each marker, consistent with the finding of Win et al, 2004 on *cyt b* gene [13], [17]. Comparisons of *cyt b* nucleotide sequences revealed 10 different substitutions between the variant and classical *P. ovale* types, one of which was a non synonymous mutation, M248I (Table 1). Comparisons of the classical and variant *cox1* nucleotide sequences, also revealed 10 different mutations, one of which was a non synonymous mutation M211I (Table 2). Comparisons of *ldh* classical and variant *P. ovale* nucleotide sequences showed 13 different substitutions, two of which were non synonymous mutations, S143P and K168N (Table 3).

**Table 4:** Human *P. ovale* strains, strain code, geographical location of origin, nucleotide sequence, type and GenBank accession number.

Species	Strain code	Origin	GenBank accession number *cytb*	GenBank accession number *cox1*	Type
*P. ovale*	5894	Angola	FJ409567	FJ409571	classical
*P. ovale*	CAMBO	Cambodia	FJ409567	FJ409571	classical
*P. ovale*	3044	Republic of Central Africa	FJ409567	FJ409571	classical
*P. ovale*	5979	Ivory Coast	FJ409567	FJ409571	classical
*P. ovale*	3149	Gabon	FJ409567	FJ409571	classical
*P. ovale*	4646	Guinea	FJ409567	FJ409571	classical
*P. ovale*	3740	Democratic Republic of Congo	FJ409567	FJ409571	classical
*P. ovale*	4419	Cameroon	FJ409566	FJ409570	variant
*P. ovale*	5401	Madagascar	FJ409566	FJ409570	variant
*P. ovale*	2132	Mali	FJ409566	FJ409570	variant
*P. ovale*	5994	Mali	FJ409566	FJ409570	variant
*P. ovale*	2668	Rwanda	FJ409566	FJ409570	variant
*P. ovale*	3043	Zimbabwe	FJ409566	FJ409570	variant

The sequences presented are derived from a single PCR-sequencing event. The differences observed between these sequences, though likely to reflect reality, might be the result of PCR amplification artefacts.

**Figure 1:**
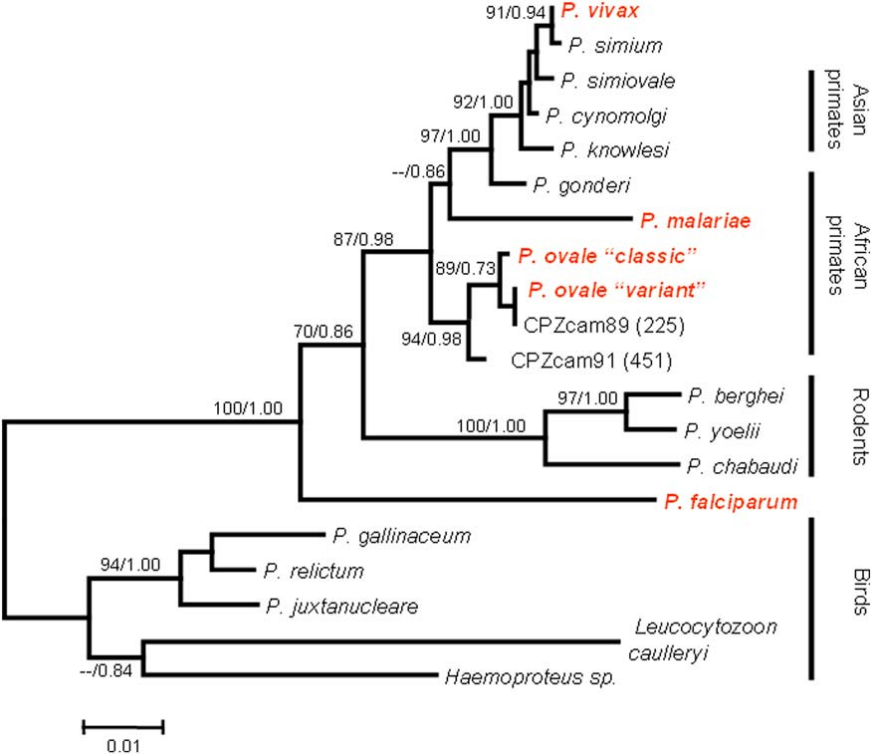
Phylogeny of Haemosporidia inferred from *cyt b* and *cox1* nucleotide sequences. Values are bootstrap percentages obtained by maximum likelihood analysis (left of the slash, values under 70% not shown) and Bayesian posterior probabilities (right of the slash, values less then 0.7 not shown), *P*. = *Plasmodium*. In red: Human malaria parasite species. Usual hosts are presented on the right side.

Both of the methods used, maximum likelihood (ML) and Bayesian analyses, produced the same tree topology consistent with previous published *Plasmodium* phylogenetic analysis [18], [19]. The phylogenetic relationships between the two *Plasmodium* strains isolated from chimpanzees to classical and variant *P. ovale* types, and the position of these strains within primate parasite group, are presented in Figure 1. The two chimpanzee parasites formed a monophyletic group with the two human *P. ovale* types. Monophyly was well supported by Bayesian posterior probabilities of 0.98 and a bootstrap value of 94%.

## Discussion

The characterization of 13 *P. ovale* human isolates, using mitochondrial *cyt b* and *cox1* markers and nuclear *ldh* marker from 12 different geographical locations, confirmed the diversification of human strains of *P. ovale* into two types, classical and variant [13].

We reported here the first molecular finding of three chimpanzee *Plasmodium* isolates, one (CPZcam89 (225)) genetically identical to *P. ovale* variant type, one other (CPZcam91 (451)) closely related to human *P. ovale* types and a third one (CPZcam63 (2360)) showing mixed infection composed of *P. reichenowi* and *P. ovale* related parasite (the latter exhibits an *cyt b* sequence identical to CPZcam91 (451) *cyt b* sequence parasite). Phylogenetic analyses inferred from *cyt b* and *cox1* concatenates are well supported and show a monophyletic group composed of human *P. ovale* types and related chimpanzee parasites. The monophyly of the group is confirmed using *ldh* nuclear partial gene sequences (data not shown).

*P. schwetzi* has been originally described by Reichenow in 1920 in blood apes in Cameroon [15]. *P. schwetzi* is morphologically similar to both *P. vivax* and *P. ovale* parasites that infect humans, and to date there are two equally convincing arguments to favour one or the other of these species as the most closely related to *P. schwetzi* [15]. Experimental infections by *P. schwetzi* in humans have also been reported [20] and in 1970, Contacos established its potential as a zoonosis for Africa [21]. At present, no isolate of this parasite from which molecular sequences can be obtained is available.

*P. schwetzi* often occurs as a mixed infection with *P. reichenowi* and *P. rodhaini*, the two other African great ape *Plasmodium* species described morphologically similar to *P. falciparum* and *P. malariae* respectively. In this study, we found one chimpanzee co-infected with *P. reichenowi* and a *P. ovale* related parasite molecularly identical to CPZcam91 (451) isolate. The CPZcam91 (451) chimpanzee parasite might be identified as being *P. schwetzi* regarding reports available on this species. Nevertheless, there is not enough evidence to support this. Morphological and other molecular information are needed to establish the identity of this parasite.

The identical sequences of CPZcam89 (225) chimpanzee parasite strain to the *P. ovale* variant type on both mitochondrial *cyt b* and *cox1* and nuclear *ldh* markers suggest possible cross-species transmission between human and chimpanzee hosts in Cameroon. Interestingly, a prevalence of *P. ovale* higher than that usually reported in Africa (above 10%) has been reported in two villages in the Manyemen forest province in Cameroon, where humans and great apes live in sympatry [6]. Furthermore, earlier, Lysenko and Beljaev (1969) previously reported a close relationship between *P. ovale* prevalence in humans and proximity to great apes in Africa [12].

No direct evidence for human malaria parasite transmission between apes and humans was reported in Gabon [22], but natural transmissions of human malaria parasites to non human primates have been reported in South America. *P. falciparum*, *P. vivax* and *P. malariae* transmissions to wild monkeys of the rainforest in French Guyana [23] and to Brazilian wild monkeys [24] have also been documented. Experimental transmission of *P. ovale* to chimpanzees via sporozoite inoculation has been reported [25].

This study provides the first evidence of human *P. ovale* variant type in chimpanzees in Cameroon. A large molecular epidemiology study would be required to improve the documentation of potential natural bidirectional transmission between chimpanzee and human populations living in sympatry, making it possible to evaluate the potential role of African great apes as a reservoir for *P. ovale* in West Africa. The question raised by Haydon et al. (2002) concerning the possibility of human *Plasmodium* species being permanently maintained in chimpanzee populations, from which infection is transmitted to human, remains to be explored [26].

## Materials and Methods

### Chimpanzee and gorilla DNA specimens

Chimpanzees and gorillas, originated from different areas of Cameroon, were, for the most part, initially kept as pets for a variable period of time and then either brought to the local zoos or sanctuaries or confiscated by the Ministry of Environment and Forestry, then gathered in captivity. These animals were sampled and included during virological studies lead by the Virology Unit of Centre Pasteur du Cameroon [27], [28]. A DNA bank was constituted between 1998 and 2004.

In total, we tested 130 DNA samples from great apes for *Plasmodium* infection, using *cytochrome b* (*cyt b*) molecular tools: 105 chimpanzees from 4 subspecies (60 *Pan t*. *troglodytes*, 39 *Pan t*. *vellerosus*, 3 *Pan t*. *schweinfurthii* and 3 *Pan t*. *verus*), 8 chimpanzees of undetermined subspecies and 17 gorillas (*Gorilla gorilla*).

Detailed information on the three positive samples: CPZcam89 (225): *Pan t*. *troglodytes* subspecies, juvenile female, collected in February 2000; CPZcam 91 (451): *Pan t*. *troglodytes* subspecies, adult male, collected in February 2001; CPZcam63 (2360): *Pan t*. *vellerosus* subspecies, adult male, collected in September 1998.

### *Cyt b* PCR amplification

We amplified 708 bp *Cyt b* gene fragments with two sets of primers, one for PCR reaction, PLAS1 (5′-GAGAATTATGGAGTGGATGGTG-3′) and PLAS2a (5′-GTGGTAATTGACATCCWATCC-3′) and one for nested-PCR, PLAS3 (5′-GGTGTTTYAGATAYATGCAYGC-3′) and PLAS4 (5′-CATCCWATCCATARTAWAGCATAG-3′) [29].

These primers are specifics for Haemosporidia parasites and do not amplify DNA from other Apicomplexa parasites or host DNA. PCR and nested-PCR were carried out in a final volume of 25 μl, under the following conditions: 2.5 μl of each primer (10 pmol/μl), 2 mM of each dNTP, 0.5 U of *Taq* polymerase (Solis), 2 mM MgCl_2_ and 2 μl of DNA, heating for 5 minutes at 94°C, 30 s at 94°C, 30 s at 55°C and 1 min 30 s at 72°C for 40 cycles and a final extension phase for 10 minutes at 72°C. The PCR products were sequenced by Macrogen (Korea) using PLAS3 and PLAS4 primers.

The parasites isolated from African great apes were also characterized molecularly by another gene, the *cytochrome c oxidase 1* gene (*cox1*). This mitochondrial gene has been chosen for the international barcoding programme for biodiversity identification [30]. Like *cyt b*, it is a conserved gene and is useful for resolving phylogenetic relationships between populations of parasite species that have diverged over tens or hundreds of millions of years [31], [32].

### *Cox1* PCR amplification

We amplified 964 bp *Cox1* gene fragments with the PCR primer set, *cox1a*: 5′-CGCCTGACATGGATGGATAATAC -3′ and *cox1b*: 5′-CCATTTAAAGCGTCTGGATAATC -3′ and the nested-PCR primer set, *cox1c*: 5′-GATTAACCGCTGTCGCTGGGACTG -3′ and *cox1d*: 5′-CGTCTAGGCATTACATTAAATCC -3′.

These primers are specifics of Haemosporidia parasites and do not amplify DNA from other Apicomplexa parasites or host DNA. PCR and nested-PCR were carried out in a final volume of 25 μl, under the following conditions: 2.5 μl of each primer (10 pmol/μl), 2 mM of each dNTP, 0.5 U of *Taq* polymerase (Solis), 1.5 mM MgCl_2_ and 2 μl of DNA, 5 minutes at 94°C, 30 s at 94°C, 30 s at 53°C for PCR and 30 s at 58°C for nested-PCR, and 2 minutes at 72°C for 40 cycles, with a final extension period of 10 minutes at 72°C. The PCR products were sequenced by Macrogen (Korea) using *cox1c* and *cox1d* primers.

The nuclear lactate dehydrogenase (*ldh*) gene has also been used to characterize parasites isolated from chimpanzees.

### *Ldh* PCR amplification

We amplified 350 bp *ldh* gene fragments with two sets of primers, one for PCR reaction, LDH1 (5′-GGNTCDGGHATGATHGGAGG-3′) and LDH2 (5′-GCCATTTCRATRATDGCAGC-3′) and one for nested-PCR, LDH7 (5′-TGTDATGGCWTAYTCVAATTGYMARGT-3′) and LDH8 (5′-CCATYTTRTTNCCATGWGCWSCDACA-3′) [17].

These primers are specifics for Haemosporidia parasites and do not amplify DNA from other Apicomplexa parasites or host DNA. PCR and nested-PCR were carried out in a final volume of 25 μl, under the following conditions: 2.5 μl of each primer (10 pmol/μl), 2 mM of each dNTP, 0.5 U of *Taq* polymerase (Solis), 2,5 mM MgCl_2_ and 2 μl of DNA ,heating for 5 minutes at 94°C, 30 s at 94°C, 30 s at 55°C for PCR and 30s at 52°C for nested-PCR, and 1min at 72°C for 40 cycles and a final extension phase for 10 minutes at 72°C. The PCR products were sequenced by Macrogen (Korea) using LDH7 and LDH8 primers.

### *P. ovale* human strains

We also characterized *P. ovale* from 12 isolates collected from 11 different African locations and 1 isolate collected from South-East Asia, Cambodia (Table 4), in collaboration with the National Reference Center for Malaria (AP-HP, Hôpital Bichat-Claude Bernard, Paris, France) using the *cyt b*, *cox1* and *ldh* partial gene sequences.

### Phylogenetic analyses

The *cyt b*, *cox1* and *ldh* sequences were checked using chromatograms and CLUSTALW alignment to ensure that none of the positions was ambiguous [33]. Mixed infection was discarded from the phylogenetic study. Phylogenetic analyses were based on the use of 708 bp *cyt b* and 964 bp *cox1* concatenated sequences (Table 5). Reference sequences without ambiguous positions for either *cyt b* or *cox1* were retrieved from GenBank.

**Table 5:** Parasite taxa, with host name, geographical location and GenBank accession number of the *cyt b* and *cox1* sequences used for the phylogenetic analysis

Parasites	Host	Geographical location	GenBank accession number *cyt b*	GenBank accession number *cox1*
*P. falciparum*	*Homo sapiens*	Tropical regions	M76611	M76611
*P. gonderi*	Old World monkeys	Central Africa	AY800111	AY800111
*P. knowlesi*	Old World monkeys	Malaysia	AY598141	AY598141
*P. malariae*	*Homo sapiens*	Tropical and subtropical regions	AF069624	AF182848
*P. vivax*	*Homo sapiens*	Tropical and subtropical regions	AY598139	AY598139
*P. simiovale*	Old World monkeys	Asia	AY800109	AY800109
*P. simium*	New World monkeys	South America	AY800110	AY800110
*P. cynomolgi*	Old World monkeys	Southeast Asia	AY800108	AY800108
*P. ovale* classical	*Homo sapiens*	Tropical regions	FJ409567	FJ409571
*P. ovale* variant	*Homo sapiens*	Tropical regions	FJ409566	FJ409570
CPZcam89 (225)	*Pan t. troglodytes*	Tropical regions	FJ409565	FJ409569
CPZcam91 (451)	*Pan t. troglodytes*	Tropical regions	FJ409564	FJ409568
*P. yoelii*	*Thamnomys rutilans*	Central Africa	M29000	M29000
*P. berghei*	*Grammomys surdaster*	Central Africa	AF014115	AF014115
*P. chabaudi*	*Thamnomys rutilans*	Central Africa	AF014116	AF014116
*P. gallinaceum*	*Gallus gallus*	Vietnam	AB250690	AB250690
*P. relictum*	Birds	North America	AY099032	EU254593
*P. juxtanucleare*	*Gallus gallus*	Asia	AB250415	AB250415
*Leucocytozoon caulleryi*	Birds	Tropical regions	AB302215	AB302215
*Haemoproteus sp.*	*Lichenostomus frenatus*	Australia	AY733087	AY733087

Statistical analysis, based on the Xia and Xie method, was conducted to examine whether the number of substitutions was saturated or not [34]. In this method, both transitions and transversions were plotted against evolutionary distances calculated with the JC69 model. The relative rates at which transitions and transversions saturated at the third position were compared by counting substitutions in all pairwise comparisons between sequences. The analysis showed that the third base was saturated, and this base was therefore discarded for subsequent phylogenetic analyses.

We identified the most appropriate nucleotide substitution model, based on hierarchical likelihood ratio tests (hLRTs), Akaike Information criterion (AIC) and bayesian information criterion (BIC) values, using PHYML [35] in a similar way to Modeltest [36]. The Hasegawa, Kishino and Yano statistic HKY [37] was favoured by the hLRT and BIC tests. Rate variation between sites was allowed, with a gamma distribution for four rate categories for the nucleotide and amino acid data, allowing for invariant sites. Maximum likelihood and Bayesian trees were inferred using the previously described model. Maximum likelihood (ML) analysis was carried out with Phyml [38], with nodal robustness evaluated by non-parametric bootstrapping (1000 replicates). Bayesian analysis was performed with MrBayes [39], using two runs of 1 million generations sampled every 100 generations. Convergence was determined using the standard deviation of the split frequencies and runs were stopped when a value of less than 0.01 was reached. The burn in phase was defined as the first 250,000 generations.
